# Impact of Anodal and Cathodal Transcranial Direct Current Stimulation over the Left Dorsolateral Prefrontal Cortex during Attention Bias Modification: An Eye-Tracking Study

**DOI:** 10.1371/journal.pone.0124182

**Published:** 2015-04-24

**Authors:** Alexandre Heeren, Chris Baeken, Marie-Anne Vanderhasselt, Pierre Philippot, Rudi de Raedt

**Affiliations:** 1 Laboratory for Experimental Psychopathology, Psychological Science Research Institute, Université catholique de Louvain, Louvain-la-Neuve, Belgium; 2 Department of Psychiatry and Medical Psychology, Ghent University, Ghent, Belgium; 3 Department of Experimental Clinical and Health Psychology, Ghent University, Ghent, Belgium; Bournemouth University, UNITED KINGDOM

## Abstract

People with anxiety disorders show an attentional bias for threat (AB), and Attention Bias Modification (ABM) procedures have been found to reduce this bias. However, the underlying processes accounting for this effect remain poorly understood. One explanation suggests that ABM requires the modification of attention control, driven by the recruitment of the dorsolateral prefrontal cortex (DLPFC). In the present double-blind study, we examined whether modifying left DLPFC activation influences the effect of ABM on AB. We used transcranial direct current stimulation (tDCS) to directly modulate cortical excitability of the left DLPFC during an ABM procedure designed to reduce AB to threat. Anodal tDCS increases excitability, whereas cathodal tDCS decreases it. We randomly assigned highly trait-anxious individuals to one of three conditions: 1) ABM combined with cathodal tDCS, 2) ABM combined with anodal tDCS, or 3) ABM combined with sham tDCS. We assessed the effects of these manipulations on both reaction times and eye-movements on a task indexing AB. Results indicate that combining ABM and anodal tDCS over the left DLPFC reduces the total duration that participants’ gaze remains fixated on threat, as assessed using eye-tracking measurement. However, in contrast to previous studies, there were no changes in AB from baseline to post-training for participants that received ABM without tDCS. As the tendency to maintain attention to threat is known to play an important role in the maintenance of anxiety, the present findings suggest that anodal tDCS over the left DLPFC may be considered as a promising tool to reduce the maintenance of gaze to threat. Implications for future translational research combining ABM and tDCS are discussed.

## Introduction

**Fig 1:**
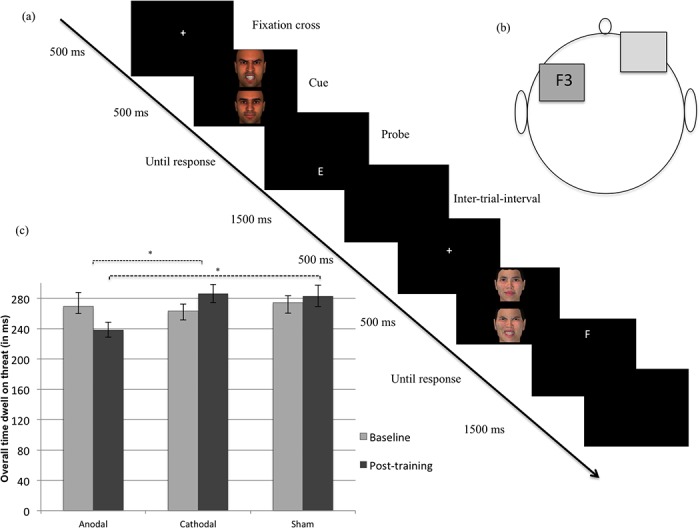
Illustration of the attention bias modification procedure designed to reduce the attentional bias towards threat, the tDCS setup, and the change in the duration time that participants’ gaze remained fixated on threat as a function of time and condition. Part a: In the original version of the dot-probe paradigm, participants viewed two stimuli (i.e., a threatening and a neutral) presented in two areas of a computer screen for approximately 500 ms. Immediately after the pictures disappeared, a probe replaced one of the stimuli. Participants responded to the probe as quickly as possible. In attention training, researchers typically modify the original task such so that the probe nearly always (i.e., 95% of the trials) replaces the neutral stimulus, thereby training subjects’ attention to be redirected towards non-threat cues. To avoid the publication of images depicting individuals without their written informed consent (as outlined in the PLOS consent form), the faces shown in the illustration are not from the Karolinska Directed Emotional Faces but are 3D-computer generated faces. Part b: One electrode was positioned centered over F3 (left DLPFC) and the other was placed over the contralateral supraorbital area. In the anodal condition, the electrode positioned centered over was the anode and the other one the cathode. In contrast, the reverse allocation was used in the cathodal condition. For the sham stimulation, the electrodes were positioned similar as when administering tDCS stimulation; however, the current was ramped down after 30 seconds. In each condition, a constant current of 2 mA intensity was applied for 25 min. Part c: Mean duration time that participants’ gaze remained fixated on threat at baseline and post-training. Error bars represent standard errors of the mean. *, *p* <. 05 (corrected using the Tukey procedure).

Over the two last decades, evidence has accumulated that individuals who suffer from anxiety disorders, regardless of the type of anxiety, exhibit an attentional bias (AB) for threatening stimuli (for a meta-analysis, see [1]), specifically concerning impaired disengagement of attention from threat such as angry faces [2–4]. Recently, researchers have started to investigate the causal nature of these biases in the *maintenance* of anxiety disorders by directly manipulating AB. Using a modified version of the dot probe paradigm (see Fig 1a), it has been observed that training anxious individuals to attend to non-threat (i.e., attention bias modification; ABM) reduces AB, which, in turn, reduces anxiety [5–6]. At a fundamental level, these findings support a central tenet of several cognitive models of anxiety disorders, i.e. that information-processing biases may causally maintain the disorders [7]. However, uncertainty still abounds regarding the mechanisms that mediate this effect. Several explanations have been proposed to account for the mechanisms underlying such a plasticity of AB and, in turn, its impact on anxiety.

The most likely explanation focuses on top-down attention control (AC), which is necessary when one needs to regulate attentional allocation [8–10]. As suggested by Bishop’s model [11–12], AB might result from a failure to recruit top-down AC, and this failure is associated with decreased activation of the left dorsolateral prefrontal cortex (DLPFC).

Accordingly, neuroimaging studies have demonstrated that anxious individuals exhibit a reduced activation of the left DLPFC during inhibition tasks [11]. Moreover, in line with the hypothesis that DLPFC activation (as a proxy of AC) is involved in ABM, it has been demonstrated that inducing AB for threat is related to altered activation of the left DLPFC to emotional stimuli rather than to change in subcortical regions [13]. Notwithstanding this preliminary evidence, this causal hypothesis can only be tested by directly manipulating the recruitment of the left DLPFC *during* ABM procedures. Such a manipulation can be achieved by combining transcranial Direct Current Stimulation (tDCS) with ABM. tDCS consists of the application of a weak (0.5–2 mA), direct electric current through electrodes positioned over one’s scalp which are able to reach the neuronal tissue and induce polarization-shifts on the resting membrane potential [14]. Anodal stimulation facilitates cortical activity, whereas cathodal tDCS has opposite effects.

Very recently, Clarke and his collaborators [15] provided the first experimental evidence that a direct increase of activity in the left DLPFC influences the effects of ABM. In this study, participants received either anodal or a sham (placebo) tDCS while completing an ABM procedure designed to induce an AB either towards or away from threat. While the participants who received sham stimulation during ABM did not show any significant change in AB from baseline to post-training, those receiving anodal tDCS evidenced a change in AB in the targeted direction (towards or away from threat). Because Clarke and colleagues intended to either increase or decrease AB, they conducted their experiment among a sample of undergraduates with mid-level trait-anxiety scores. This way, they aimed to decrease the likelihood that the participants recruited for their study already possessed a strong AB towards or away from threat. As a consequence, whereas Clarke and collaborators are the first to establish the causal influence of left DLPFC activation on AB plasticity through ABM, it is uncertain whether these results can generalize towards high-anxious individuals. This is important given that high-anxious individuals are usually those targeted by ABM procedures. Therefore, clarifying the impact of the neuromodulation over the left DLPFC during ABM in a sample of highly trait-anxious individuals is the critical next step in the translation of neuroimaging (correlational) research on ABM [13] to neuromodulatory (causal) interventions that aim to improve the effects of ABM procedures [15].

Additionally, Clarke and colleagues only examined the combination of ABM and anodal tDCS. These data provide indications that the activation of the left DLPFC is indeed a facilitator of AB plasticty through ABM procedures, but it does not mean that this region is necessarily causally implied in ABM. Further evidence regarding the underlying function of the DLPFC during ABM can only be achieved by downregulating the activity of the left DLPFC during ABM. In other words, individuals receiving cathodal tDCS to downregulate the left DLPFC activity during an ABM procedure designed to reduce AB to threat should demonstrate a significantly weaker reduction in AB than those receiving sham tDCS during such a procedure.

Moreover, it has been argued that many of the usual reaction time (RT) measures of AB may not be the most optimal index of the attentional processes [16,17]. Indeed, RT measures only reveal a snapshot of attention at a single point in time [17,18], and are consequently not optimal for detecting multiple shifts of attention that typically occur within a typical 500 ms stimulus duration period [17–19]. In contrast, eye-tracking may provide a valuable complementary measure during RT tasks given that this technology provides a continuous measure of visual attention [16–19]. In addition, whereas RT measures during the dot-probe task merely provide an indication of the direction of attention, eye-tracking allows distinctions between initial orienting (reflected in saccade sequences) from subsequent dwell time (reflected in fixation duration) [17].

Hence, the aim of the present study was to examine the influence of tDCS over the left DLPFC during an ABM procedure (designed to reduce AB to threat) on AB in a selected sample of highly trait-anxious individuals with eye-tracking measurements. Based on the above-mentioned AB studies [11–13,15] as well as on prior tDCS studies looking at top-down AC processes (for a meta-analysis, see [20]), we decided to modulate the left DLPFC. Participants were randomly assigned to one of three conditions tDCS condition during an ABM procedure designed to reduce AB to threat: 1) cathodal tDCS, 2) anodal tDCS, or 3) sham tDCS. As the main outcome of the present study was AB, we assessed the effects of these conditions on both RT and EMs indices during a dot-probe task, administered before and after the ABM procedure. We hypothesized that if a change in AB through an ABM procedure designed to reduce AB is facilitated by an increased activity within the left DLPFC, then those participants who receive anodal tDCS during such ABM procedure should demonstrate stronger reduction in both RT and EMs indices of AB. On the other hand, if ABM requires left DLPFC activation, then participants who receive cathodal tDCS during the ABM procedure should demonstrate a significant weaker reduction in AB as compared to the participants receiving sham tDCS during the ABM procedure. Regarding EMs indices, given that this study is the first to examine the impact of ABM on EMs, several hypotheses can be formulated. Because several prior eye-tracking studies reported that high-anxious individuals exhibit a facilitated gaze orientation towards threat [21,22], one possibility is that change in AB would be mainly reflected through modification of this biased gaze orienting towards threat. Alternatively, since several authors have suggested that poorer AC (as a proxy of DLPFC deactivation) may modulate the maintenance of attention to threat [8–10,23] and that ABM might be effective by reducing this exacerbated maintenance to threat [2,4], anodal tDCS during the ABM procedure may thus reduce the duration of the participants ‘gaze fixation on threat.

## Method

### Ethics Statement

Participants were provided with full details regarding the aims of the study and the procedure. All participants gave their written informed consent. The CONSORT checklist is available as supporting information (S1 Checklist). The study was approved by the Ethical Committee of the University Hospital of Ghent University (UZGent), and carried out according to the 1964 Declaration of Helsinki.

### Participants

**Fig 2:**
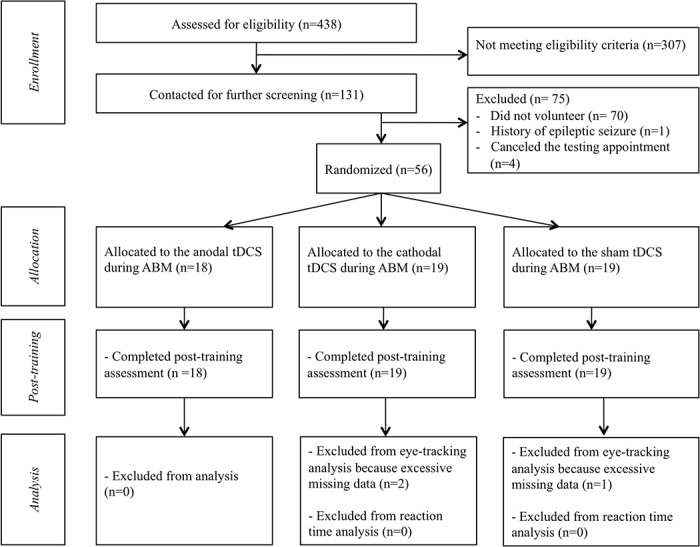
Flowchart depicting passage of participants through the study. ABM is for Attention Bias Modification; tDCS is for transcranial Direct Current Stimulation.

We recruited 56 right-handed Caucasian female students with elevated trait-anxiety scores, with a mean age of 19.91 (*SD* = 1.79, *Min* = 18, *Max* = 25). Participants were drawn from a pool of 438 female undergraduates at Ghent University based on their score (*M* = 37.53, *SD* = 9.33, *Min* = 20, *Max* = 73) on the trait-version of the State and Trait Anxiety Inventory (STAI; [24]). As depicted in Fig 2, among the 438 participants, the 30% of the sample (n = 131) with the more elevated scores on the trait version of the STAI (*M* = 57.11, *SD* = 6.15, *Min* = 40, *Max* = 73) were contacted for further screening and were invited to the UZGent hospital for testing. Additionally, the inclusion criteria were that the participants: (a) had no current/history of psychiatric disorder, using the International Neuropsychiatric Interview (M.I.N.I.; [25]), (b) no current/history of neurological problems or implanted metal objects over the head, (c) no current psychotropic medications, (d) had normal or corrected-to-normal vision, and (e) were not pregnant (at this end, all the participants were submitted to a pregnancy test at the beginning of the experiment). Of the 131 participants, one was discarded as she reported history of epileptic seizure and 60 accepted our invitation to volunteer for participation. Of these 60 participants, four canceled their testing appointments. The remaining 56 participants were included in the study (trait version of the STAI: *M* = 47.54, *SD* = 9.11, *Min* = 40, *Max* = 73). Their characteristics are listed in Table 1. Because, in this experiment, we extended the study of Clarke and his collaborators [15] to individuals with elevated trait-anxiety, trait-anxiety scores of the current study and the study of Clarke and collaborators were compared. Participants in the current study did report significantly higher trait-anxiety scores as compared to the participants in the Clarke et al.’s study [*t* = 7.05, *p* < 0.001, *d* = 1.25].

**Table 1:** Participant characteristics (*SD* in parentheses).

	Anodal tDCS during ABM (N = 18)	Cathodal tDCS during ABM (N = 19)	Sham tDCS during ABM (N = 19)
Age ^**NS**^	19.89 (1.88)	20.00 (1.70)	19.84 (1.89)
BDI-II ^**NS**^	7.89 (6.85)	9.68 (8.25)	6.68 (5.45)
STAI-S ^**NS**^	51.94 (3.89)	51.21 (2.82)	51.42 (2.99)
STAI-T ^**NS**^	47.79 (9.05)	47.47 (8.99)	47.37 (9.77)
RRS ^**NS**^	22.56 (8.88)	22.83 (7.15)	23.53 (5.23)

ABM is for Attention Bias Modification; tDCS is for transcranial direct current stimulation; BDI-II is for the Beck Depression Inventory Second Edition; STAI-S is for the State version of the State and Trait Anxiety Inventory; STAI-T is for the Trait version of the State and Trait Anxiety Inventory; RRS is for the Ruminative Response Scale; NS is for Non-significant.

### Measures

#### Control measures

Complementarily to the screening measurements, validated self-completion questionnaires were used to assess depression (Beck Depression Inventory 2^nd^ Edition, Beck: BDI; Steer, & Brown, [26]) and state- and trait-anxiety (State and Trait versions of the STAI; [24]), and ruminations (Ruminative Response Scale: RRS; [27]). The BDI-II is a self-report measure of symptoms of depression. The STAI is a self-report measure of anxiety. While the STAI-Trait measures anxiety proneness, the STAI-State assesses the individual’s current level of anxiety. The RRS is a measure of rumination. In the present experiment, the validated Dutch versions of these scales were used (for the BDI-II, see [28]; for the STAI, see [29], for the RRS, see [30]).

#### RT-based measurement of AB

RT index of AB was assessed using the dot-probe task [31]. Because the standard ABM procedure relies on a modified version of the dot-probe discrimination task (see below), we used a detection version of the task to prevent potential practice effect when assessing AB throughout the experiment [32,33]. This task consisted of 96 trials delivered in one block. Each trial began with a central fixation cross that appeared on screen for 500 ms. Immediately following the disappearance of the cross, two faces of the same person, one neutral and one angry facial expressions, appeared on the screen for 500 ms. One face appeared to the left of the screen, whereas the other face appeared to the right of the screen. Immediately following their disappearance, a probe (i.e., “X”) replaced one of the faces. The probe remained on the screen until the participant indicated the location (right versus left) of the probe by pressing a corresponding button. The inter-trial interval was 1500 ms. The task was programmed using E-Prime 2 Professional (Psychology Software Tools, Pittsburgh, PA, USA). Participants were asked to press one of the two response buttons as quickly and accurately as possible to classify the probe as appearing left or right. They were instructed to look at the fixation cross at the start of each trial. We used an equal number of trials in each condition as a function of emotional face location (left or right) and probe location (left or right). Stimuli consisted of 24 different face pairs (12 male, 12 female), each pair displaying neutral-angry facial expressions, randomly selected from the Radboud Faces Database [34]. Each pair appeared four times representing all combinations of the locations and probe types (96 trials = 24 faces pairs x 2 faces position x 2 probe position). The same material and task were used at baseline and post-training. Each of the 96 trials appeared in a different random order for each participant and each time of assessment (baseline and post-training). Pictures were 1024 pixels high, 680 pixels large, and were separated by 160 pixels.

#### Eye movement indices of AB

EMs were tracked during the dot-probe task (see above) with a 300-Hz Tobii (TX300) eye tracker (Tobii Technology AB; Falls Church, VA, USA). This system consists of 23-inch computer screen with a camera and infrared LED optics embedded beneath it to record EM based on the corneal reflection caused by the infrared light source. Prior to start the task, participants first began with a two-dimensional calibration of the eye-tracking system in which participants were asked to fixate a visual marker that appeared at nine different locations on the screen in random order (in the four corners of the screen as well as midway in between these locations and the screen center). Following this, a validation cycle verified that the EM measurement was consistent and accurate to nearest 0.3 degree of visual angle. The time course of the visual inspections was measured during the whole dot-probe task, with the system recording the position of the eyes at a rate of 300 Hz with an accuracy of 0.4 degrees and a precision of 0.14 degrees of visual angle while participants binocularly viewed the stimulus presentation.

### Experimental manipulations

#### tDCS over the left DLPFC

Direct electrical current was applied by a saline-soaked pair of surface sponge rubber electrodes (35 cm2) and delivered by a battery-driven stimulator (Neuroconn, Ilmenau, Germany). Given our specific interest for the left DLPFC, one electrode was positioned centered over F3 according to the 10–20 international system for electroencephalogram electrode placement and the other was placed over the contra lateral supra orbital area (see Fig 1b). This electrodes placement and method of DLPFC localization is in accordance with prior tDCS studies over the left DLPFC looking at AC processes (for a meta-analysis, see [20]). In each condition, a constant current of 2 mA intensity was applied for 25 min. In the anodal condition, the anodal electrode was positioned over the left DLPFC and the cathode was positioned over the right supra orbital area. The reverse electrode montage was used in the cathodal condition (i.e. anodal over right supra orbital area, cathodal over left DLPFC). For sham stimulation, the electrodes were positioned similarly as when administering tDCS stimulation; however, the current was ramped down after 30 seconds. This procedure is commonly used by tDCS researchers and has been found to be an almost optimal and reliable placebo condition [14]. Because we included anodal and cathodal stimulation in the present study, the anodal electrode montage was used for a half of the participants who received sham stimulation, whereas the cathodal electrode montage was used for the other half. Condition assignment was determined using 60 five-digit codes and two letters (i.e., A and B; each referring to one of the two montages). These codes were selected among the 200 five-digit codes database for double-blind study mode provided with the DC-stimulator (Neuroconn, Ilmenau, Germany). They were randomly ordered on a list assigning a five-digit code and a letter to each participant. Prior to each tDCS stimulation, the experimenter applied the montage associated to the letter and entered the five-digit code into the tDCS stimulator, which began the appropriate stimulation. As long as the study was running, this double-blind study mode was enabled and neither the participants nor the experimenter were aware of the stimulation condition. As a consequence, data collection was double-blind.

#### ABM procedure

During the neuromodulation procedure, all participants received the same ABM procedure that was designed to reduce AB to threat. The ABM procedure was based on the dot-probe paradigm modified in such a way that the probe nearly always (i.e., 95% of the trials) replaces the neutral stimulus, thereby redirecting subjects’ attention to non-threat cues (see Fig 1a). The task was programmed using E-Prime 2 Professional (Psychology Software Tools, Pittsburgh, PA, USA) and ran on a Windows XP computer with a 75 Hz, 19-inch color monitor. Each trial began with a central fixation cross (“+”) presented in the center of the screen for 500 ms. Immediately following termination of the fixation cue, two faces of the same person appeared on the screen, one face on the top and one on the bottom, with each pair displaying neutral-angry facial expressions. After the presentation of the faces for 500 ms, a probe appeared in the location of one of the two faces. Participants were instructed to indicate whether the probe was the letter E or F by pressing the corresponding arrow on the keyboard using their dominant hand. The probe remained on screen until a response was given. The inter-trial interval was 1500 ms. During each session, various combinations of probe type (E/F) and probe position (top/bottom) were presented twice (i.e., 480 = 60 face-pairs x 2 positions x 2 cue type x 2 repetitions). The stimuli were angry and neutral faces (30 males, 30 females), based on a validation [35] of the Karolinska Directed Emotional Faces [36], which is a standardized set of emotional expressions. All faces were adjusted to the same size (326 x 329 pixels).

### Procedure

Participants were randomly assigned to one of the three conditions (see above). Each participant was individually tested in a quiet room. As mentioned before, both the participants and the experimenters were blind to condition. Participants first completed questionnaires assessing their demographic characteristics and the MINI was administered by the experimenter. They completed the state and trait versions of the STAI, the BDI-II, and the RRS. Next, they were asked to complete the dot-probe task, which provided a baseline of both RT and EM indices of AB. Participants sat approximately 60 cm away from the center of the screen. Then, electrodes soaked in saline solution were placed on the participants’ scalp using the electrode montage described above. After five minutes of tDCS (including, as a function of the condition, either five minute of cathodal tDCS, five minute of anodal tDCS, or 30 seconds of sham tDCS followed by 4.5 minutes without stimulation), participants started with the ABM procedure during the remaining 20 minutes of tDCS stimulation. Participants were asked to perform the task as quickly and accurately as possible. After completing the training, participants completed the second dot-probe task to examine changes in AB. Participants were fully debriefed at the end of the study. Participants received compensation (25 euros) for their participation. All the participants were tested between January 2013 and May 2013.

## Data Preparation and Analytic Plan

### Power analysis

An *a priori* power analysis was conducted to determine the appropriate total sample size for testing hypotheses with the primary outcome variables. Based on previous meta-analysis examining the benefits of ABM among highly trait-anxious individuals [6], we expected a medium effect size of Cohen’s *f* = .25 [37]. Setting α at .05, power (1—β) at .80, and expecting a correlation of ρ = .50 between repeated measurements, the power analysis (G*Power 3.1.3; [38]) indicated that a sample size of at least 14 participants per group would yield an adequate power to detect a medium effect size.

### Data reduction

#### Behavioral performance

Based on previous studies in the field [32,39–42], data reduction for the RT performance of the dot-probe task was processed according to the recommendations of Ratcliff [43]: (1) trials with incorrect responses were excluded from the RT analyses (less than 0.28% of trials for the assessment at pre-training, less than 0.31% at post-training); (2) RT more than two standard deviations below or above each participant’s mean for each trial type were discarded as outliers (1.30% of the remaining trials at pre-training, 1.67% of the remaining trials at post-training). At both baseline and post-training, there was no significant difference among conditions regarding the number of incorrect responses and outliers [all *F*s < .60 and all *p*s > .55]. Then, we calculated a *d* (or bias) score for each participant by subtracting the mean latency when the probe appeared in the same location as the threatening face from the mean latency when the probe and threatening face appeared at different locations [39–42]. A positive bias score indicates faster detection of probes replacing threatening faces (i.e., AB for threat).

#### Gaze data

ClearView fixation filter software (Tobii Technology AB; Falls Church, VA, USA) was used to parse the sample stream of gaze data into movements (saccades) and durations. Two areas of interest (AOIs) were identified for each trial with each corresponding to the total area of one of the two faces that were presented as part of the dot-probe task. The direction of gaze, measured in degrees, was measured once every 3.33 ms. Following the previous studies that recorded EM during the dot-probe task [44,45], EM was classified as a fixation on either AOI if (a) participants were fixated in the central region (fixation cross) before picture onset for at least 100 ms, (b) EMs were stable within 1° visual angle for 100 ms or more on a defined AOI, (c) fixations were directed at either picture, rather than remaining at the central position during picture presentation. Fixations fulfilled these conditions on 87.88% of the 96 critical trials at baseline and 88.68% at post-training (participants did not fixate the fixation cross before picture onset for at least 100 ms on about 5% of the trials; a fixation was not made to either picture on 7% of the trials). These proportions were not different among conditions (all *p*s > 0.50) and were similar to those reported in previous studies using a dot-probe task [44,45]. Using these criteria, the time to first fixation (i.e. the time it took following the onset of a face pair to first fixate on a specific AOI), the first fixation duration (i.e. the duration of the first fixation) and the overall dwell time (i.e. the total duration of time that a participant’s gaze remained fixated within the boundaries of an AOI) on threat and neutral faces were derived. The time to first fixation and the duration of the first fixation gauged early or initial allocation/orienting or attention. In contrast, the gaze duration gauged the maintenance of attention. Furthermore, we also measured « the EM direction bias score », which is calculated by measuring the number of trials in which the first EM was directed towards the angry faces divided by the total number of trials with EMs to neutral or angry faces pairs [46,47]. A bias score greater than 50% indicates a preference to look at angry rather than neutral faces, while 50% reflects no bias. The decision to use these indices was based on previous studies in the field [44–47]. As done in previous studies [44–47], participants with excessive missing data were excluded from the EM analyses to avoid potential floor effects. In the present study, data from two participants (one from the cathodal condition and one from the sham condition) were excluded because fixations on the pictures were recorded on fewer than 15% of the trials. Data from another participant who was in the cathodal condition was also excluded since the gaze of this participant only fixated the central regions of the screen before picture onset on 2% of the trials. The analyses were performed on the remaining 53 participants for the EMs data. The remaining participants had recorded pictures fixations on at least 74% of the trials.

### Data analytic plan

Statistical analyses were performed using the SPSS software package (SPSS Inc., 2009). The significance level was set at 0.05, two tailed. To examine the effects of the experimental manipulation on AB, mixed linear models were used. Behavioral measurement (i.e. *d* score) as well as EM indices of AB were submitted to eight separate 3 (Group: cathodal tDCS, anodal tDCS, or sham tDCS) x 2 (Time: baseline, post-training) mixed analyses of variance (ANOVAs) with repeated measurement on the last factor. To account for multiple comparisons, we used the Benjamini-Hochberg procedure [48] to hold the false discovery rate (i.e., the expected proportion of falsely rejected null hypothesis) at 5% for the eight mixed ANOVAs we performed on the behavioral and EM indices.

We followed up significant interactions with between-group effects (independent *t*-tests) and within-group simple effects analyses (paired *t*-tests). Pairwise post-hoc comparison independent *t-*tests were corrected using the Tukey procedure.

## Results

### Group Equivalence

At baseline, all groups were similar in terms of age, *F*(2, 56) = .04, *p* = .96, BDI-II, *F*(2, 56) = .90, *p* = .41, η_p_^2^ = .03, STAI-trait, *F*(2, 56) = .10, *p* = .98, η_p_^2^ = .01, STAI-state, *F*(2, 56) = .25, *p* = .78, η_p_^2^ = .01, and RRS, *F*(2, 56) = .09, *p* = .92, η_p_^2^ < .01. Data are shown in Table 1.

### Effects of the experimental manipulation on AB

#### Behavioral data

For the *d* score, the *Group* x *Time* interaction, *F*(2, 53) = .52, *p* = .60, η_p_^2^ = .02, was nonsignificant, as was the main effect of *Time*, *F*(1, 53) = .04, *p* = .84, η_p_^2^ <.01, and the main effect of *Group*, *F*(2, 53) = .83, *p* = .44, η_p_^2^ = .03. Even if the data depicted in Table 2 suggest a difference among conditions at baseline, it should be noted that a one-way ANOVA revealed that groups did not differ on their *d* score at baseline, *F*(2, 56) = 1.03, *p* = .36, η_p_^2^ = .04. Data appear in Table 2.

**Table 2:** Means of Behavioral (d score) and Eye-movements Indices of Attentional Bias as a Function of Condition and Time (*SD* in Parentheses).

	Anodal tDCSduring ABM		Cathodal tDCSduring ABM		Sham tDCSduring ABM	
	Baseline	Post-training	Baseline	Post-training	Baseline	Post-training
*d* score	-.439 (19.49)	2.35 (17.91)	6.93 (15.36)	3.68 (11.46)	-.138 (18.36)	1.97 (7.96)
Time to first fixation on angry faces	190.60 (46.70)	179.06 (75.53)	142.94 (33.87)	154.01 (43.02)	173.45 (45.97	176.05 (53.18)
Time to first fixation on neutral faces	180.71 (36.10)	172.08 (77.04)	150.32 (49.99)	155.57 (40.53)	171.13 (49.65)	175.05 (48.01)
Duration of the first fixation on angry faces	218.79 (74.52)	241.26 (93.10)	275.39 (73.76)	266.15 (79.32)	245.16 (80.18)	228.04 (76.40)
Duration of the first fixation on neutral faces	231.10 (67.72)	249.82 (95.45)	261.09 (85.69)	267.68 (77.65)	252.47 (64.62)	248.02 (81.66)
Overall time dwelled on angry faces	269.70 (73.42)	238.54 (44.30)*	263.45 (35.25)	286.34 (53.94)	274.43 (50.72)	283.10 (61.20)
Overall time dwelled on neutral faces	257.01 (74.92)	262.79 (58.80)	257.99 (36.69)	285.46 (62.01)	287.31 (61.20)	292.54 (71.51)
EM bias score	51.20 (6.38)	52.92 (6.37)	49.68 (3.65)	49.52 (5.52)	49.24 (5.67)	52.73 (7.61)

ABM is for Attention Bias Modification; tDCS is for transcranial direct current stimulation. Group difference at post-training that was significant at *p* <. 05 (adjusted using the Tukey procedure) are flagged with “*”.

#### Gaze data

The ANOVA only showed a significant *Group* x *Time* interaction for the overall dwell time the gaze remained fixated on threat, *F*(2, 50) = 4.16, *p* = .02, ηp^2^ = .14. Neither the main effect of *Time*, *F*(1, 50) = .01, *p* = .98, ηp^2^ < .01, nor the main effect of *Group*, *F*(2, 50) = 1.69, *p* = .20, ηp^2^ = .06, was significant. This significant interaction remains significant at level .05 (with a corrected *p* at .02) after applying the Benjamini-Hochberg correction holding the false discovery rate (i.e., the expected proportion of falsely rejected null hypothesis) at 5% for the eight mixed ANOVAs we performed on the behavioral and EM indices. While participants did not differ at baseline, *F*(2, 51) = .10, *p* = .91, ηp^2^ < .01, they exhibited a significant difference among groups at post-training, *F*(2, 51) = 4.42, *p* = .02, ηp^2^ = .15. Simple effects of *Time* revealed that in the anodal tDCS group overall dwell time was reduced from baseline to post-training, *t*(17) = 2.32, *p* = .03, while those in the cathodal group, *t*(16) = 1.98, *p* = .07, or in the sham group did not, *t*(17) = 0.56, *p* = .59. As shown in Fig 1c, corrected (using the Tukey procedure) pairwise post-hoc comparison *t*-tests revealed that the mean overall dwell time on threat at post-training was significantly lower for participants who were in the anodal tDCS conditions than those who were either in the cathodal (*p* = .03) or sham conditions (*p* = .04). There were no differences between the sham and the cathodal conditions (*p* = .98).

For the other EM indices, neither the *Group* x *Time* interactions, all *F*s < 1.22 and all *p*s > .30, nor the main effects of *Time*, all *F*s < 2.80 and all *p*s > .11, or *Group*, all *F*s < 2.04 and all *p*s > .11, were significant. Data appear in Table 2.

## Discussion

The main aim of the present study was to examine the influence of tDCS over the left DLPFC during an ABM procedure (designed to reduce AB to threat) on AB in a selected sample of highly trait-anxious individuals with eye-tracking measurements. We had two mains predictions. First, we hypothesized that participants receiving anodal tDCS during the ABM procedure should demonstrate stronger reduction in both RT and EMs indices of AB than those receing sham tDCS. Second, we also predicted that participants receiving cathodal tDCS during the ABM procedure should demonstrate a weaker reduction in AB as compared to those receiving sham tDCS during the ABM procedure. Consistent with our first prediction, we observed a significant decrease from baseline to post-training in the overall dwell time the gaze remained fixated on threat for participants who received the anodal tDCS during ABM. In contrast, those who were either in the cathodal or sham conditions did not show such significant differences from baseline to post-training.

As the overall dwell time on threat refers to the maintenance of gaze to threat [17,49], the current findings of decreased dwell time suggest that an increase of the activity within the left DLPFC does facilitate the malleability of this maintenance to threat. As a consequence, our results are consistent with previous accounts pointing out that the modification of AB may be facilitated by the promotion of attention control, driven by the recruitment of the dorsolateral prefrontal cortex (DLPFC) [8–10]. However, in contrast to our second prediction, individuals receiving cathodal tDCS during ABM did not demonstrate significantly less reductions in the overall dwell time on threat than those receiving sham tDCS. Conversely, given that the participants receiving only the ABM procedure (sham stimulation) did not exhibit any change from baseline to post-training, the cathodal tDCS could not undo ABM effects in the current study. As a conquence, although the current data provide indications that the activation of the left DLPFC facilitates changes in the maintance of gaze to threat, we were not able to establish whether the activation of this region is compulsory involved in the ABM procedure. This way, the present findings are in line with previous data suggesting that the DLPFC activation may modulate the difficulty to disengage attention from threat among high-anxious individuals. For instance, highly trait-anxious individuals reporting poor AC (as a proxy of the reduced DLPFC activity) exhibit more delayed disengagement from threat [23]. Consistently, at the neural level, cortical structures centered around the prefrontal cortex and its functionally related structures (i.e., anterior cingulate cortex and orbitofrontal cortex) may mediate delayed disengagement from threat through individual differences in the ability to down-regulate the influence of limbic structures and maintain attention on task-relevant stimuli [12,50]. This hypothesis makes sense in the context of previous work demonstrating that the activation of the DLPFC is functionally related to a down-regulation of amygdala activity during the presentation of threatening stimuli [11]. Future studies should examine the impact of anodal tDCS over the DLPFC during fMRI to explore whether such activation is associated with modulation of the DLPFC—amygdala connectivity during the dot-probe task.

However, in contrast to Clarke and colleagues [15], participants receiving anodal tDCS during ABM did not show greater changes in RT indices of AB than those in the sham condition. Moreover, in striking contrast to our predictions, there were no differences in RT indices of AB from baseline to post-training for participants only receiving ABM (sham stimulation), which is, however, similar to the study of Clarke and colleagues [15]. Centrally, in contrast to previous studies using a similar single-session ABM design within the same population of highly trait-anxious individuals [5,6,51], we were unable to find any effect of ABM on RT indices of AB. There are various potential explanations for this lack of effect.

First, our measurement tools may have failed to detect change. This could be due to the low reliability of the RT indices of the dot-probe task. Indeed, studies that have examined the reliability of the RT indices of this task consistently reported very low levels of internal consistency and test-retest reliability, with all the coefficients smaller than .38 [52–54]. In this perspective, the use of a task before and after the manipulation might be problematic. Nevertheless, the impact of this weakness is limited in the present study as recent papers suggest that eye-tracking measurements are more reliable, exhibit higher temporal resolution, and are less susceptible to confounding processes than RT indices in tasks assessing AB [17]. Therefore, eye-tracking should be preferred over RTs for interpreting the data [16,17]. In the current study, reduced dwell time on threatening information was observed after anodal tDCS over the left DLPFC. This index is highly relevant in anxiety because it is related to the maintenance of gaze to threatening stimuli and to the cortico-subcortical circuitries in this psychopathology [9–12,50].

Second, it may be that the lack of RT changes result from the use of a detection version of the dot-probe task (i.e., detecting whether the X appears on left or right). Indeed, most of the previous ABM experiments used a discrimination version (i.e., discriminating between E or F). Indeed, in the detection version of the task, the participants can infer at which side of the screen the target appears by only attending one side of the screen, and thus without making attention shifts (i.e. if the probe is not in the attended location, it must by default be in the opposite position). However, given that in 87.88% of the critical trials at baseline and 88.68% at post-training participants were fixated in the central region before picture (cue) onset and EM were directed at either picture, during picture presentation, it is unlikely that the results are biased by the this problem. Moreover, the results of a former study suggest that the detection version of the task may be superior to the discrimination version [55]. Nevertheless, future studies may benefit from using a discrimination version of the dot-probe task to ensure the generalizability of the present results.

Third, it may be that the ABM training did not work properly because the participants of the current study did not exhibit an AB at baseline. In line with this suggestion, recent studies reported that the participants who demonstrated greater AB at baseline displayed significantly larger reductions in AB at post-training [32,56]. In order to test for an AB at baseline, we computed a one-sample *t*-test testing whether the *d* score at baseline significantly differed from 0 (i.e., no AB). Results show that participants in the current study did not exhibit such an AB at baseline, *t*(55) = .91, *p* > .36. Using the gaze data, we also computed a paired-*t*-tests to investigate whether gaze duration for threat significantly differ from gaze duration for neutral cues at baseline. Again, participants in the current study did not exhibit any significant difference, *t*(52) = .28, *p* = .78. Based on previous research [41], we then reran all the analyses with AB at baseline as a continuous moderator factor. The results were not significantly different. However, these results relied on post-hoc analyses, thereby limiting their validity. Moreover, it should be noted that the observation of a significant change in AB in the study of Clarke and colleagues [15] among participants who were explicitly selected to not already possess an AB tends to rule out the hypothesis that the malleability of AB *mandatorily* requires the presence of AB at baseline.

Despite the absence of effects on RT indices on the dot-probe task, the possibility to combine tDCS and ABM should not be dismissed. Indeed, the results of the current study contribute to a growing literature suggesting that AB is malleable among anxious individuals. More centrally, at a clinical level, it suggests that the maintenance of gaze to threat, as assessed here using eye-tracking device, is malleable by the combination of ABM and anodal tDCS. This finding is clinically relevant as previous works suggested that the tendency to maintain attention to threat acts as a vulnerability factor to the development and the maintenance of anxiety [2–4,57]. Further, it suggests that anodal tDCS applied over the left DLPFC may be a promising tool to reduce this maintenance of gaze to threat. As previous studies suggested that AB plays an important role in the maintenance of anxiety symptoms [5–7], developing innovative and more reliable tools for reducing AB among anxious is particularly relevant. Future studies should thus further explore the impact of anodal tDCS *per se* over the left DLPFC in the maintenance of AB and, in turn, the impact of such a reduction of AB in the maintenance of anxiety symptoms.

## Limitations

The present study has limitations. First, our participants were highly trait-anxious individuals and not clinically-diagnosed with an anxiety disorder. As a consequence, the replication of the present experiment among individuals suffering from clinically diagnosed anxiety disorders constitutes the logical next step in this translational line of research. However, although it has been widely demonstrated that anxious individuals exhibit an AB for threat regardless of the type of anxiety disorders [1,58], most of the compelling evidence regarding the efficacy of ABM procedure directly derived from studies conducted among patients suffering either from social anxiety or generalized anxiety disorders [5,6,59]. As a consequence, it remains particularly difficult to ensure that the present findings can generalize beyond these two categories of anxiety disorders. Future research should thus further examine this issue.

Second, because we did not include a condition investigating the impact of tDCS without ABM and that participants who only received ABM (sham tDCS) did not improve, we cannot exclude that the reduction of the total duration that participants’ gaze remains fixated on threat merely results from the anodal tDCS *per se* and not from the combination of tDCS and ABM. As the only previous study [15] combining tDCS and ABM did not include a condition combining tDCS and non-ABM, uncertainty still abounds. Indeed, although the findings of Clarke and colleagues [15] suggest an absence of a generic effect of tDCS because specific evidence of ABM in each direction across the different training conditions (attend threat vs. avoid threat) was found, we cannot rule out that tDCS alone might also impact on AB. As a consequence, the present results should be interpreted with caution. Future studies may benefit from designs that directly cross the presence/absence of ABM and the activation of the left DLPFC via tDCS in order to shed light on this issue.

Third, although the overall dwell time on threat can be considered as a valid measure of the maintenance of attention to threat, it cannot be considered as a solid index of attention disengagement from threat when EMs are recorded during a dot-probe task. Indeed, it has recently been argued that an accurate measure of biased attentional disengagement from threat using eye-tracking can only be correctly achieved by (1) first securing initial attention to the locus of threat stimuli prior to presenting a non-emotional neutral stimulus to a distal screen locus and (2) then assessing how the valence of the proximal information influences the shifting of eye-gaze away from it [17,18]. Consequently, future studies should explore how the present findings generalize to such a paradigm able to accurately differentiate biased attentional engagement from biased attentional disengagement.

Finally, even if our electrodes placement is based on prior studies in the field [20], the position of the reference electrode (i.e. the anode in case of cathodal stimulation and the cathode in case of anodal stimulation) may influence the overall current flow pattern through the brain, and thus the position of both electrodes should be considered [60–62]. As a consequence, future studies may benefit from ensuring that the reference electrode does not target a cortical region by using extra-cephalic placement [60,63]. However, it has been shown that this wider inter-electrode distance reduces the intensity of the stimulation under the anodal electrode [62].

## Supporting Information

S1 Checklist CONSORT Checklist. (DOC)

S1 Dataset Dataset. (TXT)
